# A Toolkit for ARB to Integrate Custom Databases and Externally Built Phylogenies

**DOI:** 10.1371/journal.pone.0109277

**Published:** 2015-01-21

**Authors:** Steven D. Essinger, Erin Reichenberger, Calvin Morrison, Christopher B. Blackwood, Gail L. Rosen

**Affiliations:** 1 Department of Electrical & Computer Engineering, Drexel University, Philadelphia, Pennsylvania, United States of America; 2 Department of Biomedical Engineering, Science, and Health Systems, Drexel University, Philadelphia, Pennsylvania, United States of America; 3 Department of Biological Sciences, Kent State University, Kent, Ohio, United States of America; Saint Louis University, UNITED STATES

## Abstract

Researchers are perpetually amassing biological sequence data. The computational approaches employed by ecologists for organizing this data (e.g. alignment, phylogeny, etc.) typically scale nonlinearly in execution time with the size of the dataset. This often serves as a bottleneck for processing experimental data since many molecular studies are characterized by massive datasets. To keep up with experimental data demands, ecologists are forced to choose between continually upgrading expensive in-house computer hardware or outsourcing the most demanding computations to the cloud. Outsourcing is attractive since it is the least expensive option, but does not necessarily allow direct user interaction with the data for exploratory analysis. Desktop analytical tools such as ARB are indispensable for this purpose, but they do not necessarily offer a convenient solution for the coordination and integration of datasets between local and outsourced destinations. Therefore, researchers are currently left with an undesirable tradeoff between computational throughput and analytical capability. To mitigate this tradeoff we introduce a software package to leverage the utility of the interactive exploratory tools offered by ARB with the computational throughput of cloud-based resources. Our pipeline serves as middleware between the desktop and the cloud allowing researchers to form local custom databases containing sequences and metadata from multiple resources and a method for linking data outsourced for computation back to the local database. A tutorial implementation of the toolkit is provided in the supporting information, S1 Tutorial. Availability: http://www.ece.drexel.edu/gailr/EESI/tutorial.php.

## Introduction

Dealing with large biological sequence datasets can be a computational burden. Unless one has access to supercomputing facilities, the scope of experimental research may be limited by in-house computational power. Fortunately, computing clusters that are easily accessible via the Internet circumvent this issue and have become an inexpensive alternative to upgrading in-house computing resources [1]. Outsourcing the heavy data crunching also enables greater flexibility in the choice of algorithm, thus enabling researchers to try novel approaches as they are published without first requiring their integration into popular desktop applications such as ARB, MacClade and DAMBE5 [2, 3, 4].

While outsourcing computation to the cloud is becoming more commonplace, it is still largely inconvenient to analyze and interpret results remotely. Popular online tools do not all-inclusively support exploratory data analysis or other tedious tasks such as alignment curation and primer design. Generalist web-based tools such as Galaxy and MG-RAST support various data processing pipelines, but have limited integrated algorithms for alignment and phylogeny [5, 6]. They also lack the rich data curation and interactive user interfaces afforded by some of the desktop applications, particularly ARB.

Our toolkit development was motivated by the need to perform massive bacterial and fungal sequence analysis. In particular, we collected genomic sequences along with pertinent metadata from multiple online repositories, aligned the sequences, constructed a phylogeny, identified clades of interest on the tree using our metadata and then designed primers for further experimental research. The datasets we employed consisted of thousands of sequences that precluded us from using local computational resources (see Results). Since our data analysis was exploratory in nature, we required a full-featured interactive application that employed a graphical user interface and possessed the computational throughput of cloud-based resources.

Table 1 highlights some of the more popular desktop and online tools potentially suited for our purpose [2, 3, 4, 5, 6, 7, 8, 9, 10, 11]. We considered software solutions that support database functions for the selection of sequences based on metadata content, alignment and phylogenetic construction capability, user interactive interfaces for tree manipulation and cost. The three leading options based on these criteria were ARB, DAMBE5, and Geneious. We chose to focus on ARB because it 1) includes a custom import data filter feature that enables the use of a user-supplied database consisting of custom tailored metadata, 2) is a popular, open-source, freely available solution with a lengthy history of use in the biological community.

**Table 1:** This table comprises a list of potential software solutions for typical genomic data analysis tasks in molecular ecology (e.g. alignment, phylogenetics, data exploration, etc.).

**Software**	**Purpose**	**Support**	**Cited**	**Cost**	**Availability**
ARB	Database Mining/Alignment/Phylogenetics/Interactive Inference	MacOS, Linux	3484	Free	http://www.arb-home.de/
DAMBE	Alignment/Phylogenetics/Interactive Inference	PC, MacOS, Linux	1574	Free	http://dambe.bio.uottawa.ca/dambe.asp
Galaxy	Alignment/Phylogeny/Metagenomics/Statistical Analysis	Web, MacOS, Linux	544	Free	http://galaxyproject.org/
Geneious	Database Mining/Alignment/Phylogenetics/Gene Annotation	PC, MacOS, Linux	933	$795	http://www.geneious.com/
MacClade	Sequence Editing/Phylogenetics/Interactive Inference	MacOS	6466	Free	http://macclade.org/
MEGA	Web-based Data Mining/Alignment/Phylogenetics/Inference	PC, MacOS	27888	Free	http://www.megasoftware.net/
MG-RAST	Metagenomic Statistical Analysis	Web	513	Free	http://metagenomics.anl.gov/
Mothur	Sequence Processing/Alignment/Clustering/Taxonomic Analysis	PC, MacOS, Linux	1419	Free	http://www.mothur.org/
PAUP*	Parsimony-based Phylogenetic Tree Building	PC, MacOS, Linux	6414	$100	http://paup.csit.fsu.edu/
Phylip	Phylogenetic Tree Building & Inference (No GUI)	PC, MacOS, Linux	19571	Free	http://evolution.genetics.washington.edu
SeaView	Alignment/Phylogenetic Tree Building & Inference	PC, MacOS, Linux	628	Free	http://pbil.univ-lyon1.fr/software/seaview

Both desktop and web-based solutions are included.

As described in the results section, the size of our dataset precluded us from performing alignment and phylogenetic tree construction locally within the ARB environment. Furthermore, we wanted to use MAFFT for our alignment algorithm, which was not supported within ARB [12]. We found the Cipres Science Gateway cloud based computing cluster, which is supported by the NSF XSEDE, to be sufficient for this task [13]. Conveniently, Cipres also supported RAxML, which we chose as the phylogenetic tree construction algorithm [14]. To complete our data analysis pipeline we developed a python toolkit to serve as the middleware between ARB on the desktop and Cipres in the cloud.

## Materials and Methods

The toolkit serves as middleware between the desktop application ARB and cloud based computing resources. It includes a set of python scripts for:

1. Forming a custom database: Extracting biological sequence and metadata information from a variety of resources.
2. Generating an ARB import filter for this custom database of sequence information.
3. Constructing alignments and phylogenetic trees remotely, importing results back to ARB for analysis and linking results with metadata in the custom database.

All python scripts and a tutorial are available at:

http://www.ece.drexel.edu/gailr/EESI/tutorial.php.

**Figure 1:**
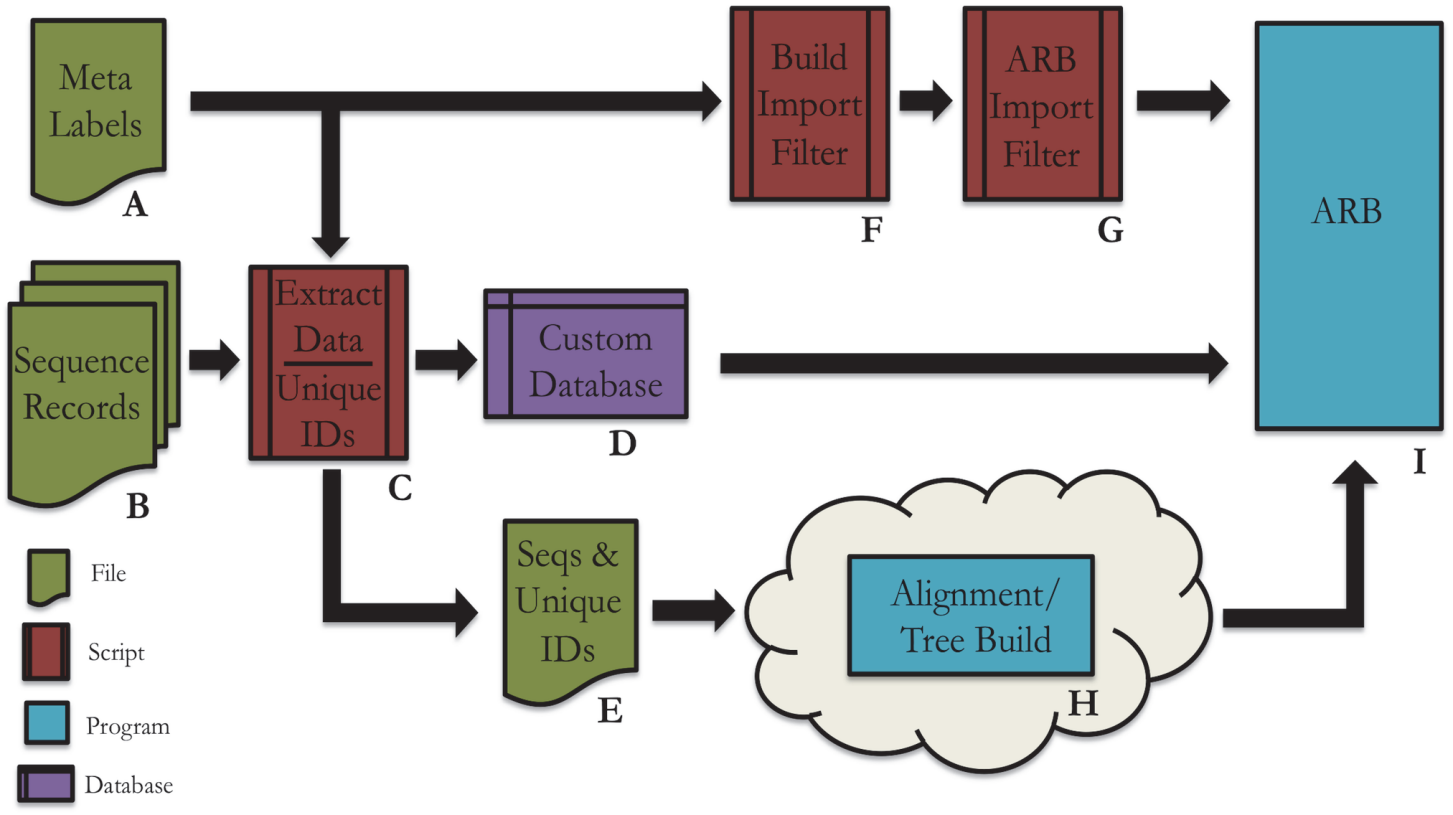
Pipeline of Toolkit: A set of steps, items and scripts for leveraging remote computational resources with local ARB installations. The scripts included methods for metadata extraction from NCBI taxonomy and GenBank files, generation of ARB import filters and assignment of unique IDs to preserve the sequence/data relationships when moving between local/remote platforms. This pipeline enables local exploration of massive datasets, while outsourcing the heavy computational alignments and tree construction.

Additionally, we have included a second example of the utility of our toolkit in the supporting information, S1 Tutorial. The standard implementation using our toolkit is shown in Fig. 1. Each step of the pipeline is now described below in greater detail.

### A: Meta-Labels

A user supplied text file containing the names of the metadata fields. For each sequence in the database, all corresponding metadata will be assigned to its respective name from this file. Metadata examples include, but not limited to: Identification (e.g. Accession, GI, PFAM, etc.), Taxonomy (e.g. Species, Genus, Phylum, etc.), Environmental (e.g. pH, Salinity, Temperature, etc.), Functional (e.g. Gene Product, Locus, Size, etc.) and Publication (e.g. Date, Author, Journal, etc.).

### B: Sequence Data

Sequence records (e.g. GenBank records) from which to extract desired sequences and metadata information.

### C: Data Extraction

A python script for extracting information from GenBank files and the NCBI Taxonomy. This script also generates a unique identification number for each database entry. The unique ID serves two purposes:

1. Alignments and phylogenetic trees constructed outside of ARB may be imported into ARB and associated with metadata.
2. Future metadata may be added to existing sequence records within the ARB environment.

### D: Custom Database

This is the FASTA formatted custom database. Each entry consists of a tab-delimited header followed by a sequence on the next line. The header information starts with the unique ID followed by all corresponding metadata, ordered according to the list of metadata names provided in the meta-labels text file described in section A.

### E: Sequences and Unique IDs

This is the sequence file for alignment and phylogenetic tree building using external algorithms and computing resources of choice. It is a FASTA formatted file containing only the unique ID in the header.

### F: Build ARB Import Filter

This is a python script for creating the import filter for the custom database. The meta-labels text file described in section A is all that is required by the script to build the filter. The script will output a file called custom_import_filter.ift.

### G: ARB Import Filter

This is the custom .ift ARB import filter for your database, which is automatically generated by the python script described above in section F. The filter must be placed into the ARB import filter directory ‘/arb/lib/import/’. To import your custom database, follow the standard ARB procedure for creating a new database.

### H: Alignment/Tree Building

The choice of tool(s) for alignment and phylogenetic tree building is at the discretion of the user. For example, you may be interested in building your tree using the maximum likelihood algorithm offered by RAxML [14], but desire to use external resources due to inadequate internal computational capabilities. The Cipres Science Gateway offers a potential solution for both sequence alignment (e.g. MAFFT) [12] and tree building (e.g. RAxML) using the XSEDE computing cluster. The user submits the sequences.fasta file from section E to Cipres and then builds a pipeline within Cipres to construct the tree. The tree output from this process would then by imported into ARB using the tree import function found under ‘Tree/Tree Admin/Import’ within ARB.

### I: ARB

This is the ARB program. Information on downloading, installation and use may be found at the ARB website: http://www.arb-home.de.

## Results

As described in the online tutorial (http://www.ece.drexel.edu/gailr/EESI/tutorial.php), our toolkit development was motivated by ongoing research to identify and explore the evolution of microorganisms that have extracellular enzymes to degrade plant polymers and transporter proteins for the uptake of polymer degradation products. We focused on particularly relevant gene families of transporters and extracellular enzymes. We required an analysis pipeline capable of processing massive datasets of fungal and bacterial sequences to look for evolutionary patterns and conserved sequence regions amenable to primer design within these gene families. Our search conducted via the NCBI [15] and PFAM [16] databases produced hundreds of thousands of sequences along with over 50 different classes of metadata, some of which are nonstandard and not easily accessible via GenBank record parsing tools (e.g. source organism). To design the primers, one may be inclined to visualize the protein product on the leaves of the tree to isolate sub-classes. All the user has to do is re-label the leaves with the protein product. Users can also re-label the leaves according to phylogeny and one of the 50+ classes of metadata. Our toolkit therefore augments ARB’s probe design and editor tools for primer design purposes. The online tutorial walks the user through a subset of our data to illustrate the use of our toolkit for aligning tens of thousands of sequences with external resources to produce a tree and then using our tools to link the associated metadata to the tree in ARB.

As a second example illustrating the utility of our toolkit, the S1 Tutorial provided with this article shows the user how they can import a curated protein super-family (e.g. Major Facilitator Superfamily (MFS) Protein Family, 440 sequences) from the NCBI Conserved Domain Database (CDD) database [17, 18] into ARB using our framework. Once imported, users now not only have access to a pre-computed alignment and phylogenetic tree but also have the power of viewing the GenBank records’ metadata on the leaves of the phylogenetic tree for further exploratory analysis in ARB. Our procedure can be applied to any protein family found in the CDD database and our framework expands the use of curated and well annotated multiple sequence alignment models provided by NCBI.

## Discussion

The described alignment and phylogenetic processing tasks are not well suited for local, non-high performance computational resources, yet users want interactive, local control over the data exploration of the results. For example, in our project, we found 10,752 sequences belonging to the Sugar Transporter protein family after accounting for duplicates. Using ARB’s Clustal W [19] accurate setting on a locally-based 2.8 GHz Apple Computer with 4G of RAM, we began to align the sequences and found that each pairwise alignment took approximately 0.019s to complete. Given that there are n*(n-1)/2 pairwise alignments required, where n is 10,752, it would take 13.29 days to complete the multiple alignment. Performing the alignment on the freely available web-based NSF funded Cipres computing infrastructure, and choosing the superior MAFFT algorithm (unavailable via ARB), the alignment completed in 14 minutes and 31 seconds when outsourced. Clearly, this is a remarkable increase in throughput and we were able to import the alignment and link it with our collected metadata in ARB in a matter of minutes. Therefore, researchers need the tools developed in our work, so that they can export computationally intensive tasks while maintaining local data exploration flexibility. After using the tools we developed, and receiving results in a matter of minutes, we were able to explore clades within our Sugar Transporter tree using various protein product metadata we had collected to aid us in our ultimate goal of choosing product-discriminative primers. If one was so inclined, with a few clicks one could re-label the tree using the publication date of the sequences to investigate the progression in which the proteins/organisms were discovered. The exploratory analysis capabilities using our toolkit are only limited by the metadata one could collect.

## Conclusion

A suite of software coupling the utility of the ARB environment with the scalable nature of computing cluster resources has been described. The software we provide allows users to outsource computationally intensive tasks while maintaining local data analysis flexibility. An example of the utility of this pipeline is provided in S1 Tutorial and S1 Scripts. All scripts, datasets, descriptions used in the pipeline and a second example of usage is provided in a tutorial at: http://www.ece.drexel.edu/gailr/EESI/tutorial.php.

## Supporting Information

S1 Tutorial A step-by-step guide implementing the toolkit.
(PDF)

S1 Scripts Toolkit code and supporting scripts for S1 Tutorial.
(ZIP)
