# Supplementary material for: A Toolkit for ARB to Integrate Custom Databases and Externally Built Phylogenies
Source: PLoS One. 2015 Jan 21;10(1):e0109277. doi: 10.1371/journal.pone.0109277 (PMC4301908; doi:10.1371/journal.pone.0109277)
Supplement: S1 Tutorial — A step-by-step guide implementing the toolkit. (PDF) [file pone.0109277.s001.pdf]

Supplementary Material

Software Requirements:
Python Version 2.7
Biopython Version 1.60
Python Modules: httplib, urllib2, ete2 (An Environment for Tree Exploration (ETE), Optional)
ARB Version 5.1

Goal: Importing alignment, phylogenetic tree and meta-data from external resources into ARB.
Motivation: Exploratory Analysis and Primer design in ARB.
Dataset: Major Facilitator Superfamily (MFS) Protein Family (440 sequences)

Available from the NCBI Conserved Domain Database (CDD)
http://www.ncbi.nlm.nih.gov/Structure/cdd/cddsrv.cgi?uid=212314
MFS: "…a large and diverse group of secondary transporters that includes uniporters, symporters, and antiporters. MFS proteins facilitate the transport across cytoplasmic or internal membranes of a variety of substrates including ions, sugar phosphates, drugs, neurotransmitters, nucleosides, amino acids, and peptides."

Procedure:

1. Download the MFS dataset from CDD. Two files: 1 for alignment and 1 for tree.
   http://www.ncbi.nlm.nih.gov/Structure/cdd/cddsrv.cgi?hslf=1&uid=cd06174&#seqhrch

   *Note: Set the Type Selection to 'top listed sequences' and the Aligned Rows to All 440 Rows before downloading the alignment file via the Structure View button.

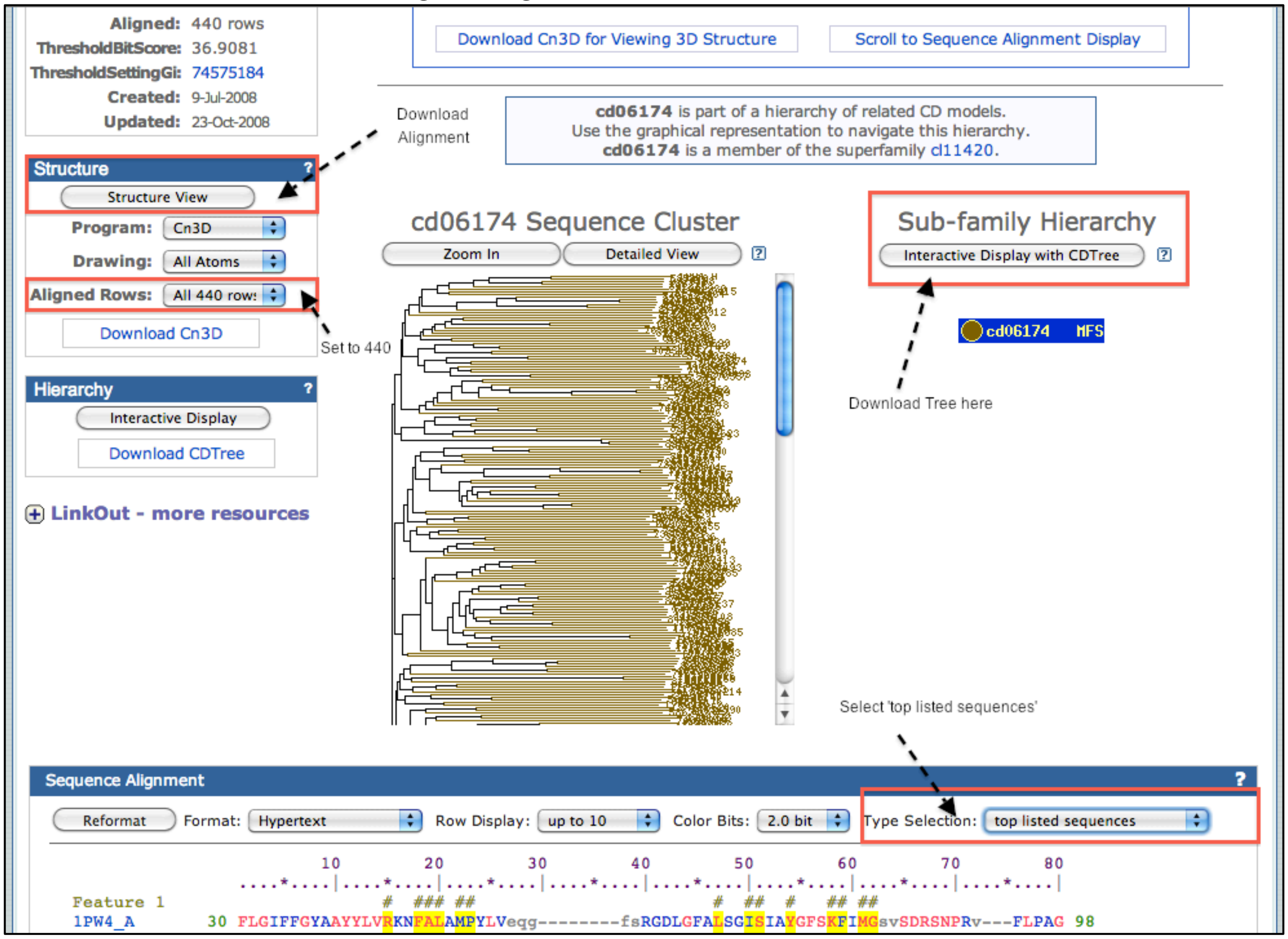

Supplementary Material

2. Download/Install CDTree tool for dataset visualization and extraction of tree.
   http://www.ncbi.nlm.nih.gov/Structure/cdtree/cdtree.shtml

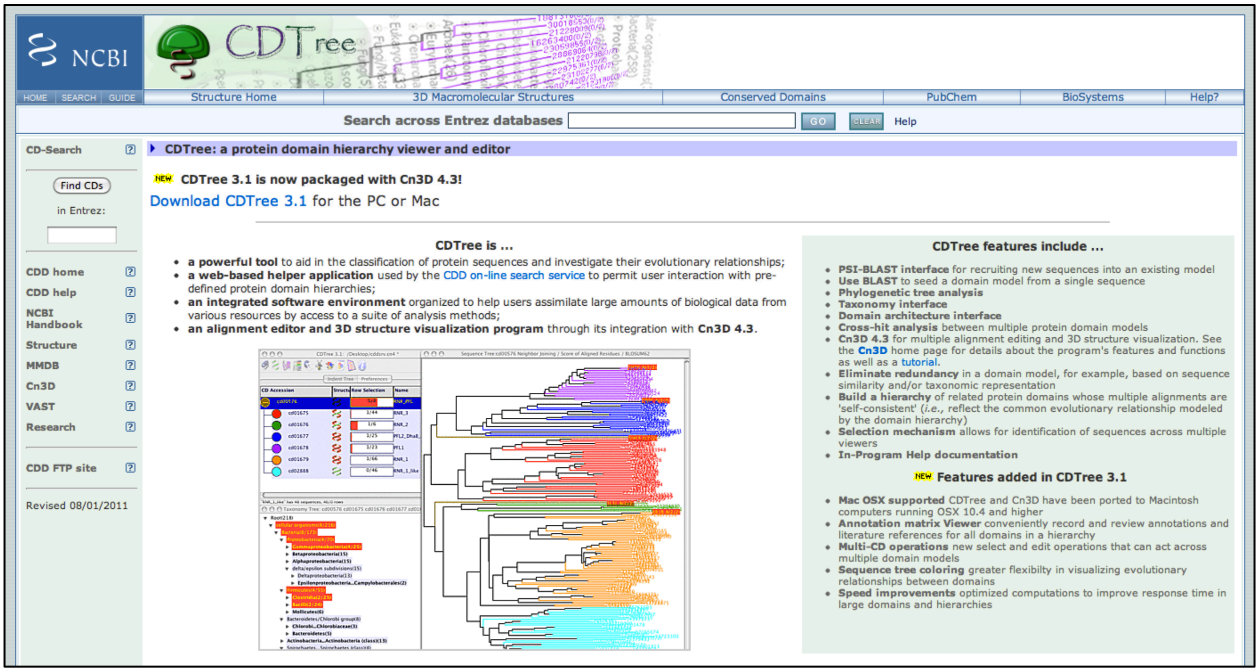

3. Open MFS dataset project (***cddsrv.cn4***). Export MFS phylogenetic tree from CDTree:
   a. Right click/Select Sequence Tree

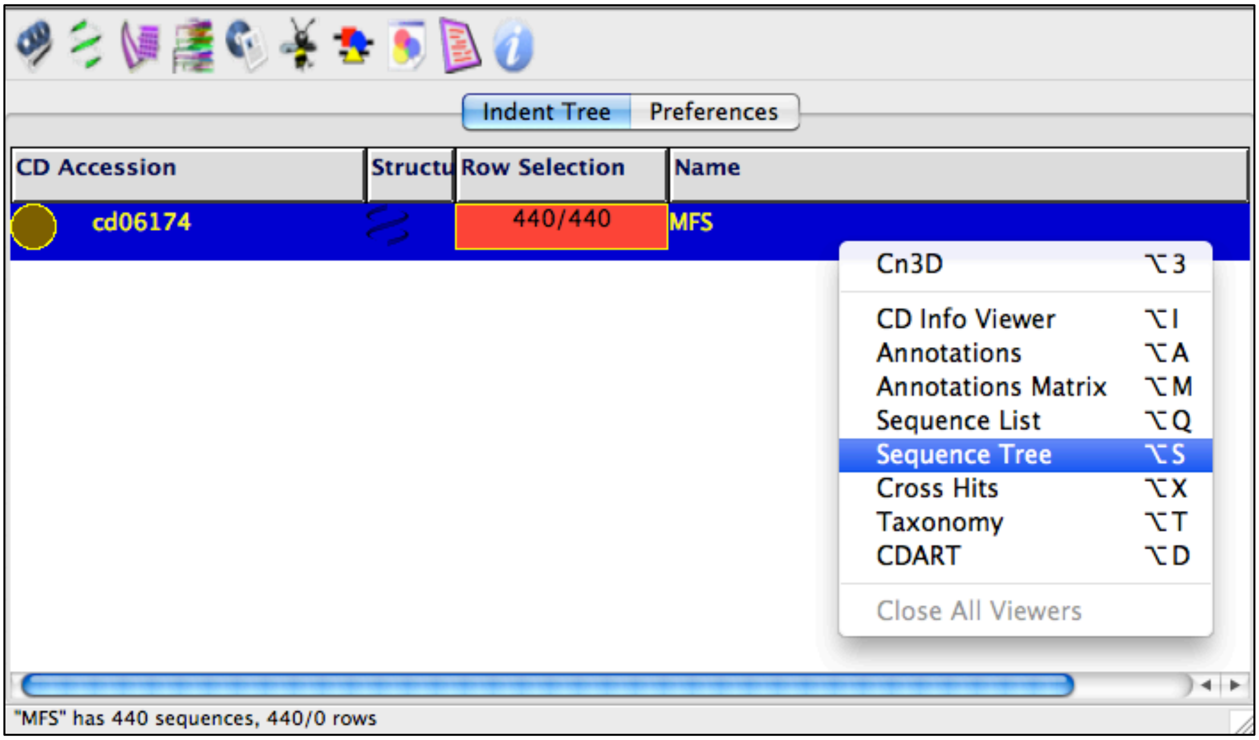

Supplementary Material

b. Create Tree

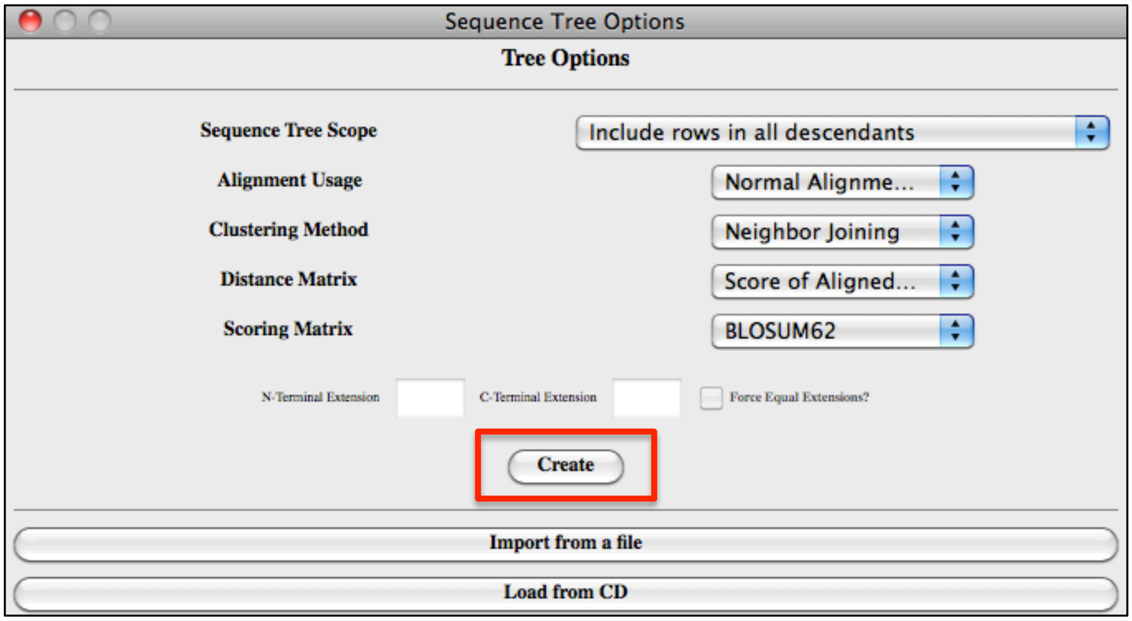

c. Export Sequence Tree: Save as ***MFS.tree***

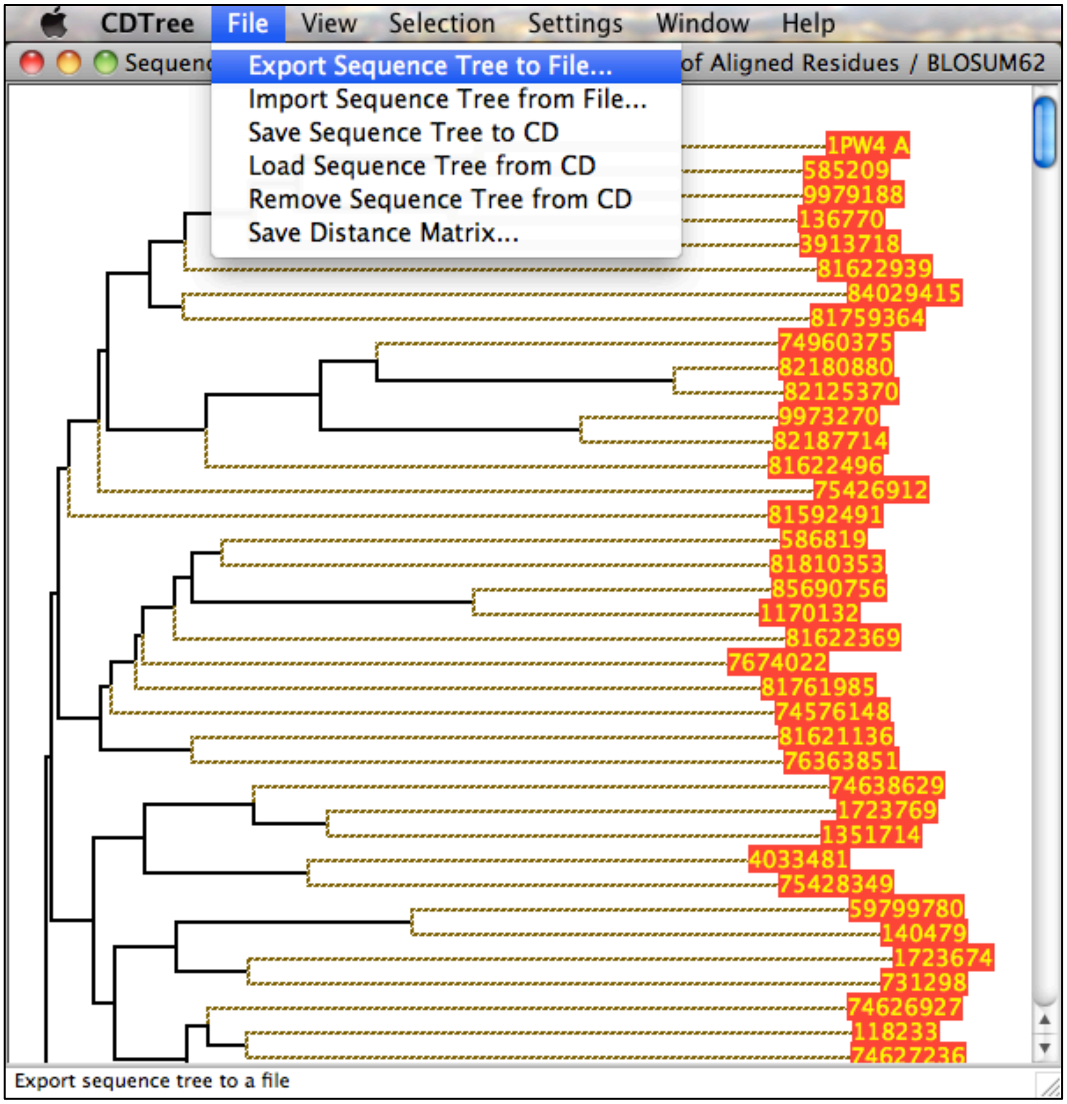

Supplementary Material

4. Download/Install/Run Cn3D tool for exporting MFS alignment.
   http://www.ncbi.nlm.nih.gov/Structure/CN3D/cn3d.shtml

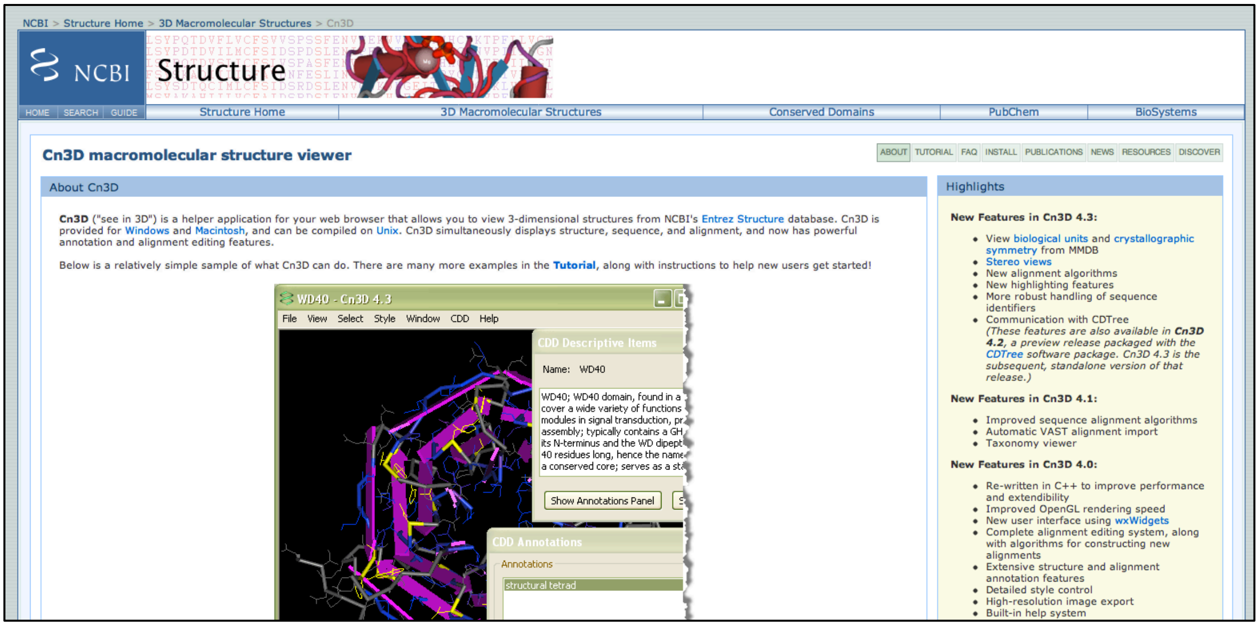

5. Open MFS dataset project (***cddsrv.cn3***). Export alignment: Save as ***MFS_Align.fasta***

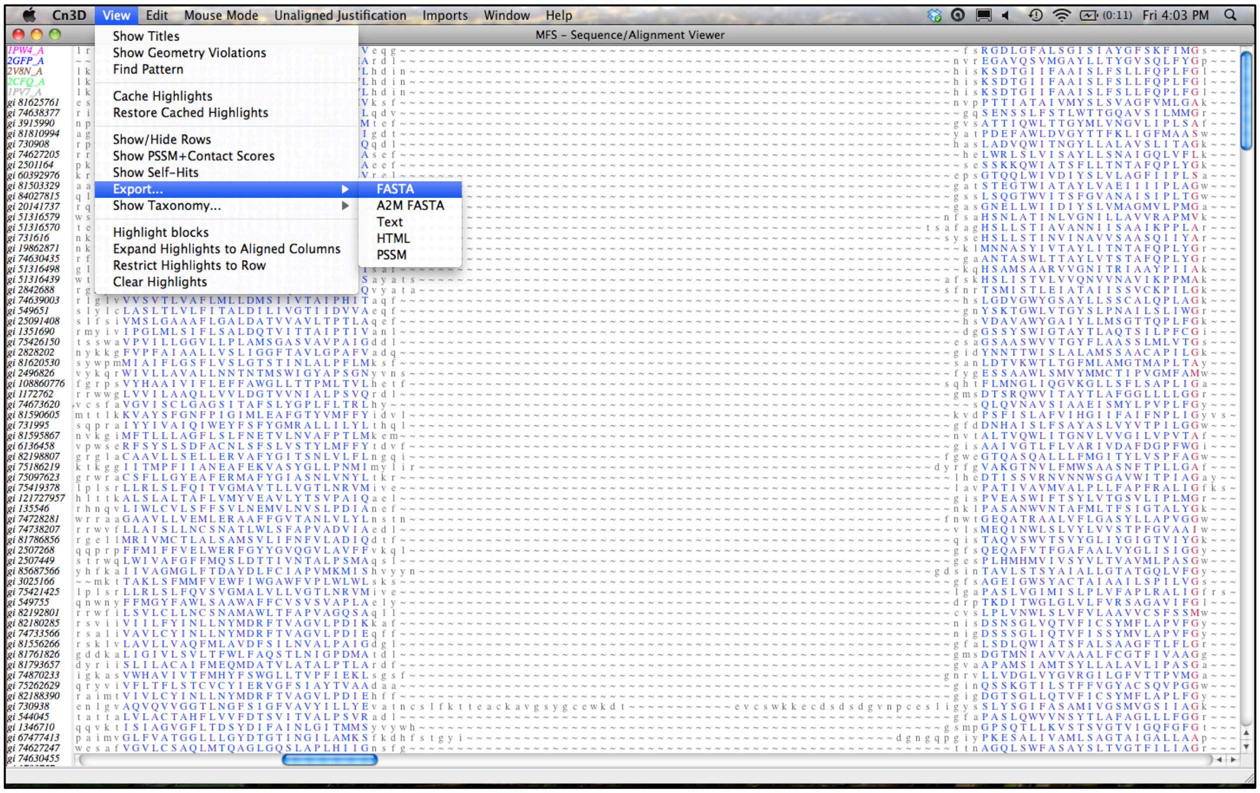

6. Assemble the Database -- Collect Meta-Data and Sequences

   From a terminal, change the directory to the ***GetDatabase*** folder. Run the script ***buildDatabase.sh*** (e.g. ./buildDatabase.sh) to obtain the GenBank files for each sequence in the dataset. Note that this script may only be run between the hours of 9pm-5am EST as per NCBI requirements. Once all of the GenBank files have been downloaded the script will proceed to construct the custom database. The completed database (***MFS_metaData.txt***) may be found at ***GetDatabase/MFS/Output/*** along with the names of the acquired meta-data fields (***MFS_Field_labels.txt***). This second file is required for building the ARB import filter described in the following section. Note that there are additional files in the Output directory that are not used as part of this tutorial.

   The ***buildDatabase.sh*** script contains a python script (***NextractGB.py***) for constructing the custom database from the downloaded GenBank files. The ***NextractGB.py*** is also capable of reconstructing the entire NCBI taxonomy and using this information to populate taxonomy field labels within the custom database. To enable this feature you must uncomment lines 89-100 in the ***NextractGB.py*** file. Note that this feature requires at least 6GB of RAM (disabled by default). The supporting files and functions for this feature is contained in the ***GetDatabase/NCBI_Taxonomy/*** directory.

   buildDatabase.sh dependencies:
   - Python version 2.7
   - Biopython version 1.60
   - Python modules httplib, urllib2

   NCBI_Taxonomy dependencies (additional):
   - taxid2lineageModule.py module (script in NCBI_Taxonomy)
   - ete2 python module (An Environment for Tree Exploration (ETE))
     - http://pypi.python.org/pypi/ete2/
       - ete2 dependencies:
         - Python Modules:
           - numpy, scipy, pyqt4, mysq, lxml

Supplementary Material

7. From the directory containing ***MFS_Field_labels.txt***, run the ***buildFilter.py*** (e.g. python buildFilter.py) script to generate the ARB import filter for the custom database. Use the ***MFS_Field_labels.txt*** as the input file and name the output, ***MFS_import_filter.ift:***

   python buildFilter.py -i MFS_Field_labels.txt -o MFS_import_filter.ift

   Place it in the ARB directory**:** ***arb/lib/import/*** (Typically, this directory is located at ***/opt/local/share/arb/lib/import/*** in OSX).

8. We need to rename the headers in the alignment file and the leaves on the tree to the respective sequence's unique ID. This will ensure that we can link the meta-data, sequences, alignment and tree all together in ARB.

   a. For the alignment:
      i. Run the ***getAccession.py*** script from the directory containing ***MFS_metaData.txt***. This will produce an alignment file (***MFS_UID.fasta***) that replaces the sequence headers with the respective unique ID. Enter the following at the prompts:
         1. Enter name of database: **MFS_metaData.txt**
         2. Enter name of alignment file: **MFS_Align.fasta**
         3. Enter desired name for Unique IDs file: **MFS_UID.fasta**

   python getAccession.py -I MFS_metaData.txt -a MFS_Align.fasta -o MFS_UID.fasta

   b. For the tree labels:
      i. We will use the Interactive Tree of Life (iTOL) to revise our tree labels. http://itol.embl.de/

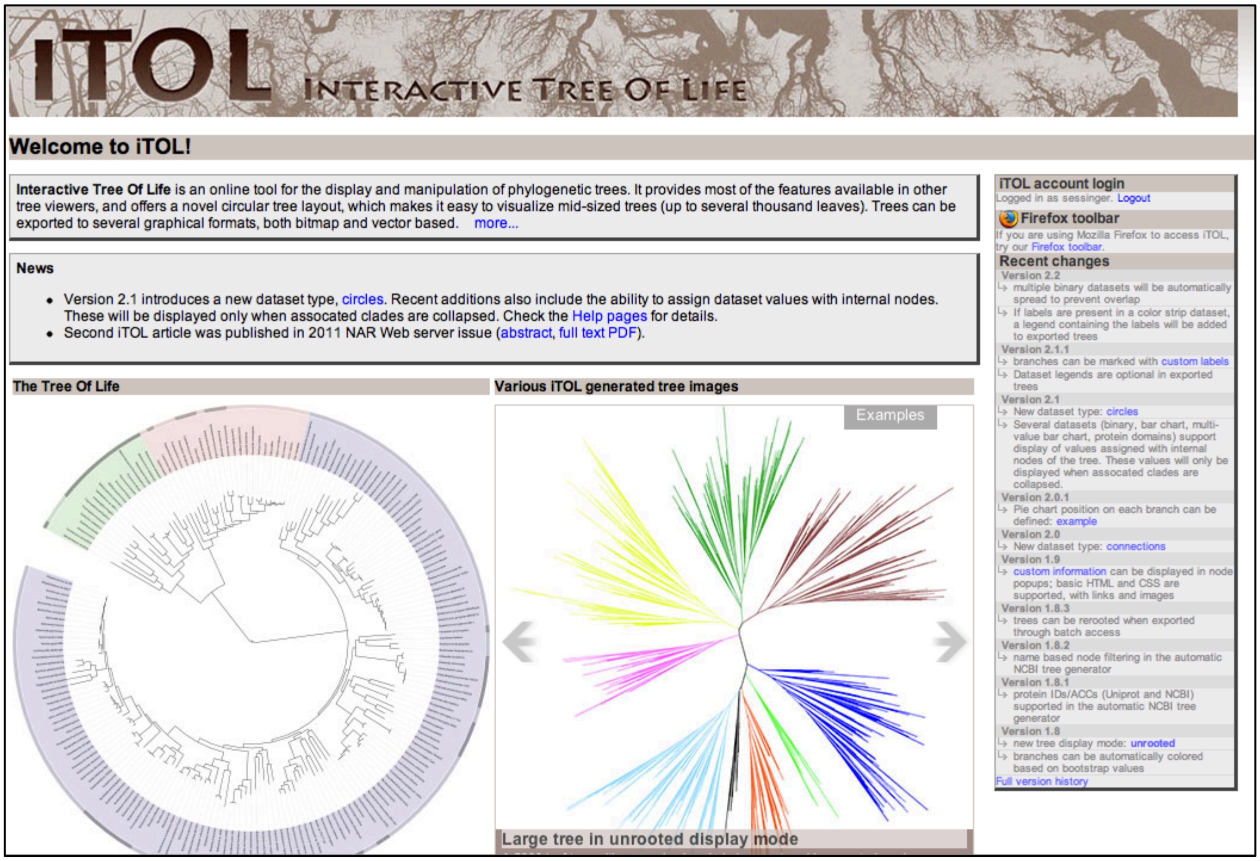

ii. Login and upload the tree file from step 3c: ***MFS.tree***

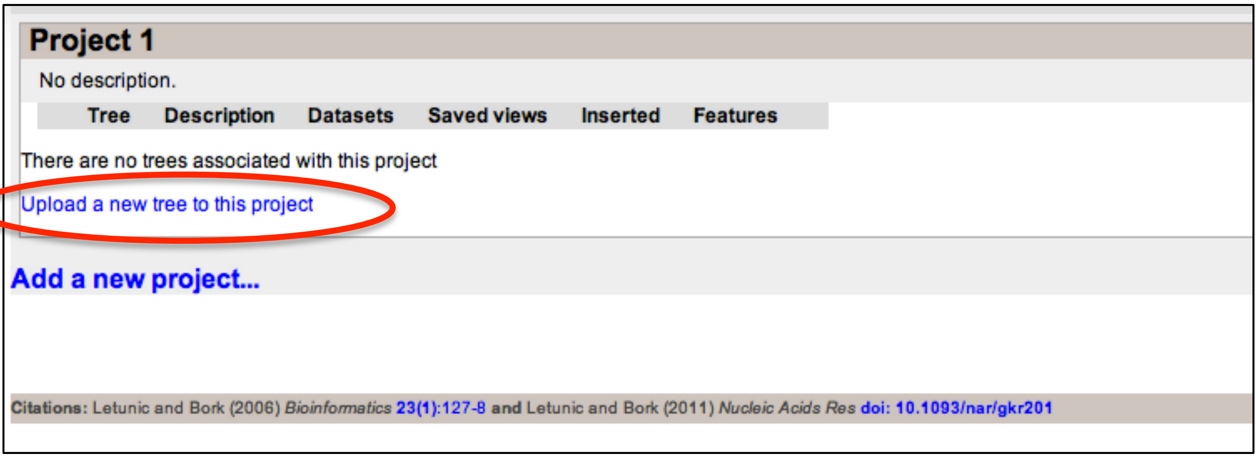

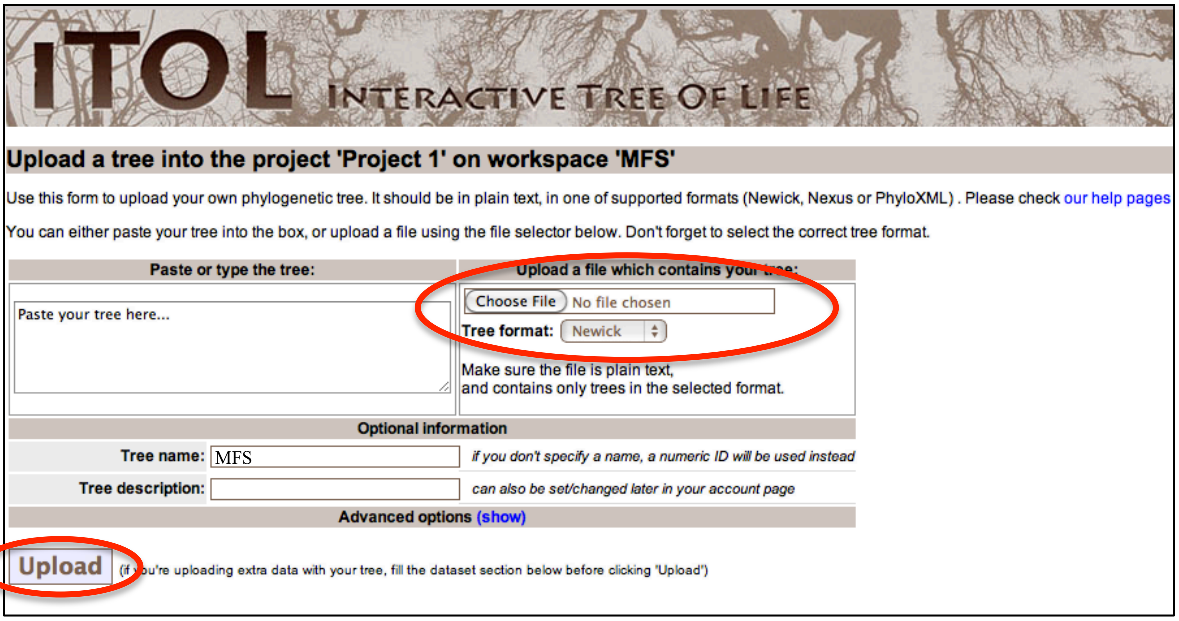

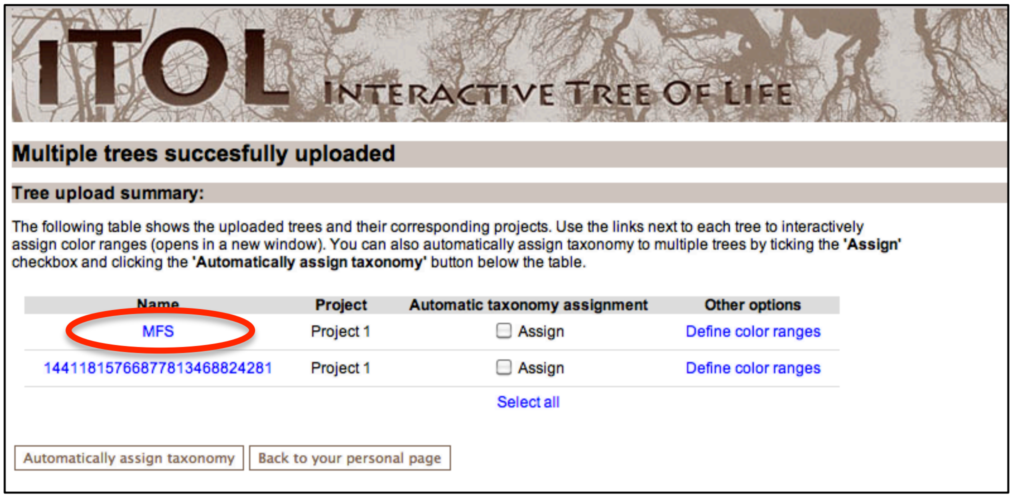

Verify tree then return back to Project 1.

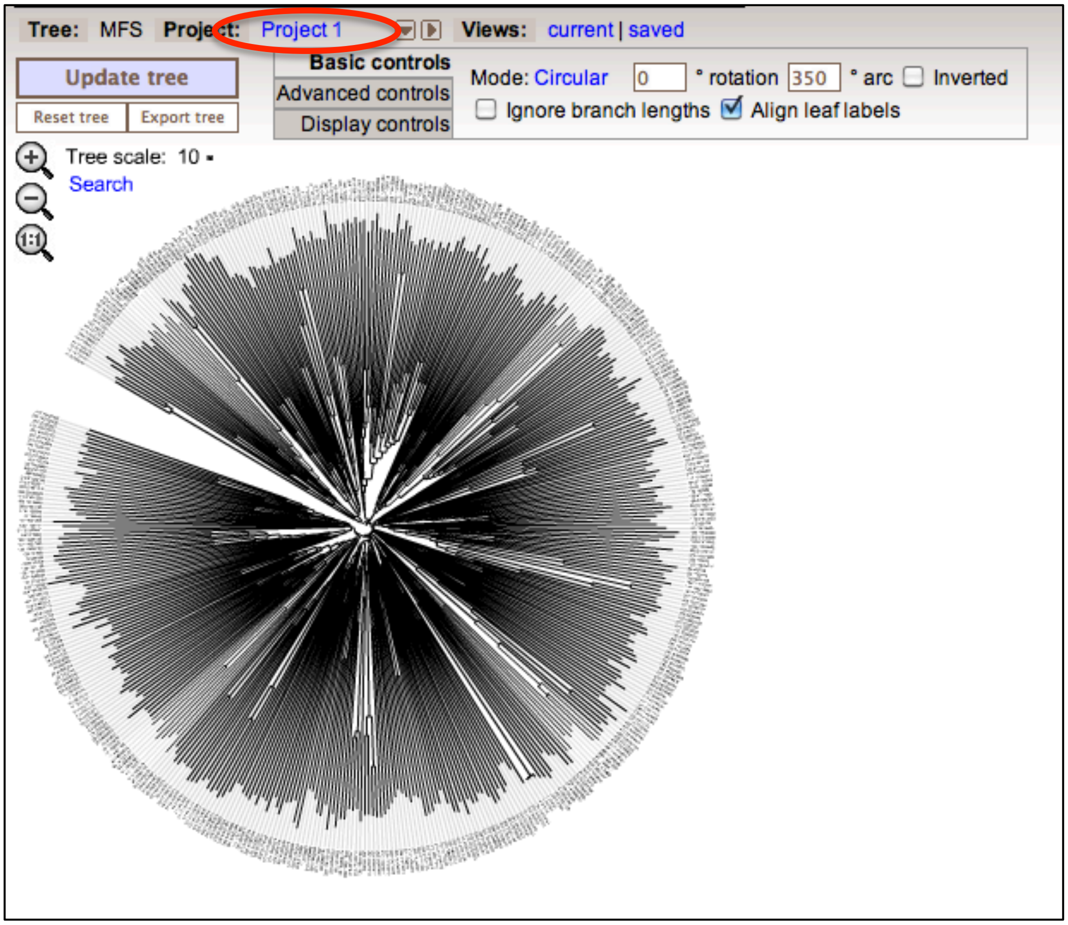

iii. Rename the leaves using the file ***TreeLabels_Mapped_New.txt***
*This file was created using the ***rename_tree_leaves.py*** script (see Appendix A).

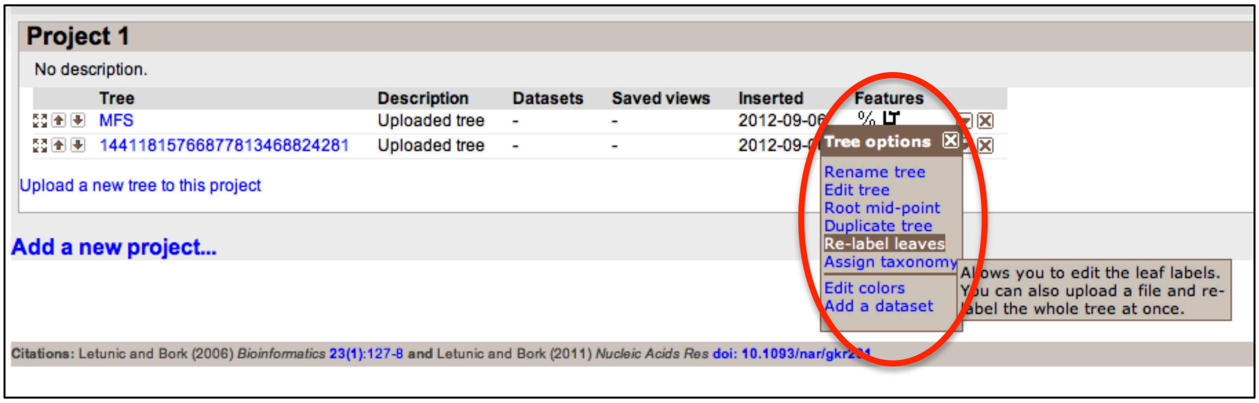

Use the file ***TreeLabels_Mapped_New.txt*** here.

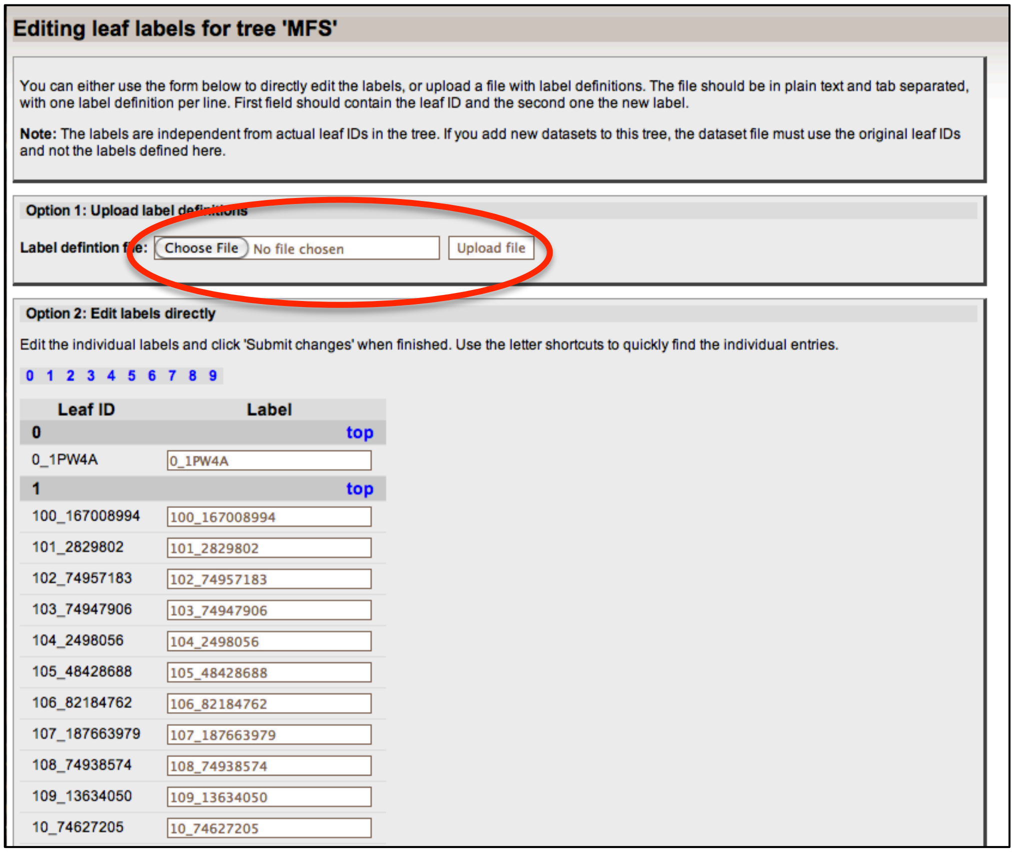

iv. Return to the tree view to export the tree with the newly assigned labels.

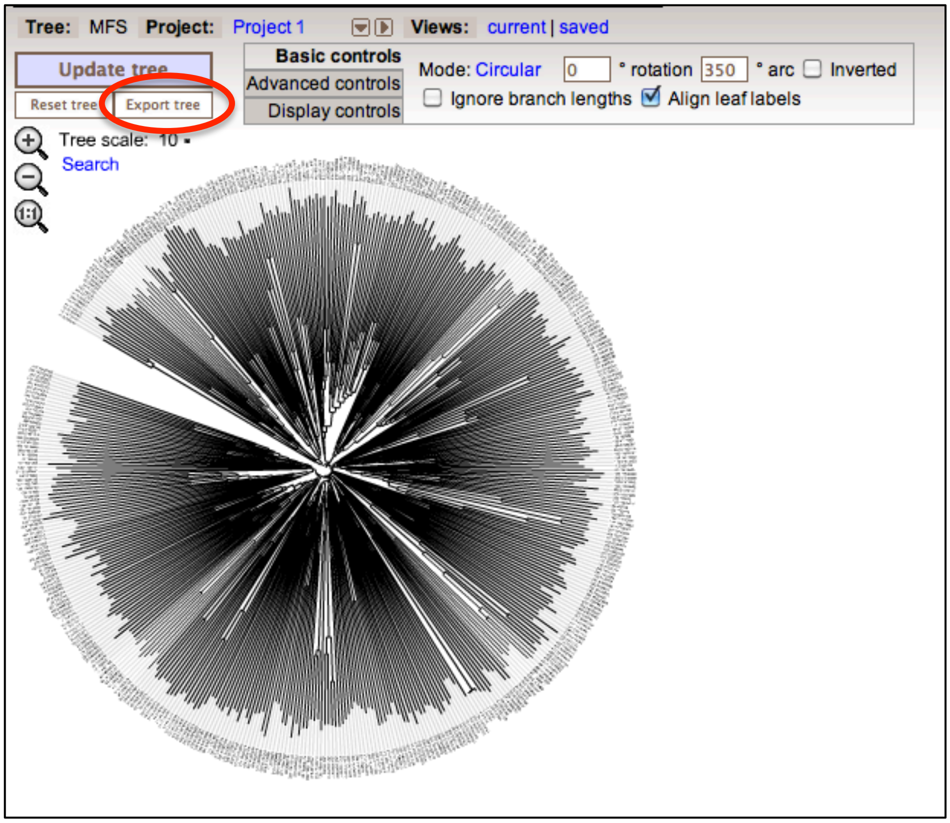

Supplementary Material

Export the tree in Newick format and save as ***MFS_UID.tree.***

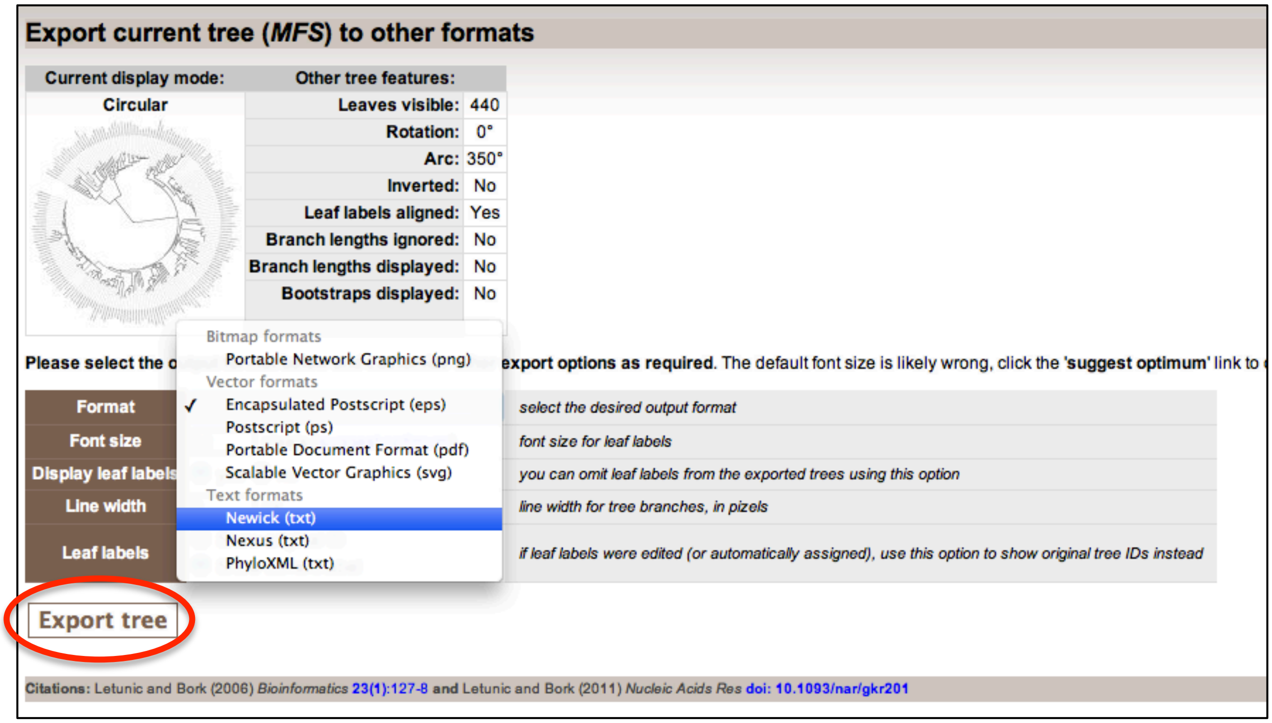

9. At this point we have four files and are ready to import them into ARB:
    a. Tree file with unique IDs: ***MFS_UID.tree***
    b. Alignment file with unique IDs: ***MFS_UID.fasta***
    c. Database file containing sequences with meta-data: ***MFS_metaData.txt***
    d. ARB import filter: ***MFS_import_filter.ift***

10. Start ARB from the command line. ARB is available at: http://www.arb-home.de/.

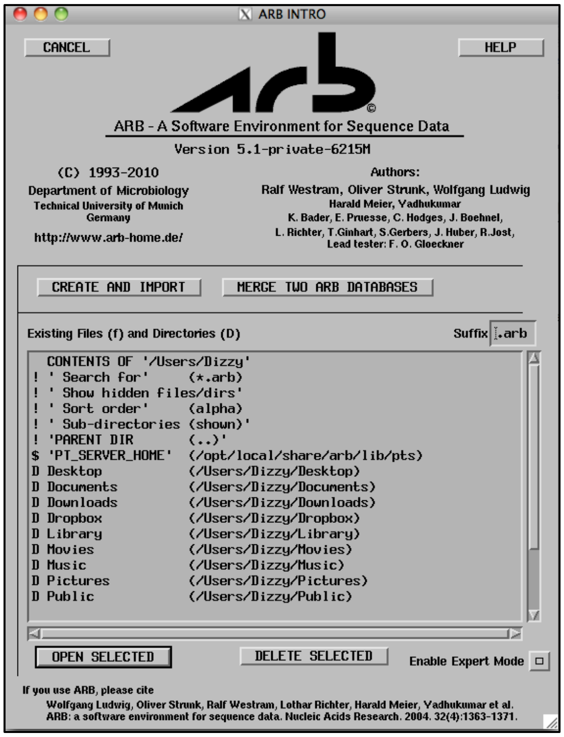

11. ***Create and Import*** the ARB database. Navigate to the directory containing the sequences and metadata ***…/MFS/Output/.*** Choose the ***MFS_metaData.txt*** file and use the ***MFS_import_filter.ift*** created in step 7. Change **Type** to **Protein**.
    Note: If prompted, select 'Format (all)'. Also, if prompted choose 'Use found names'.

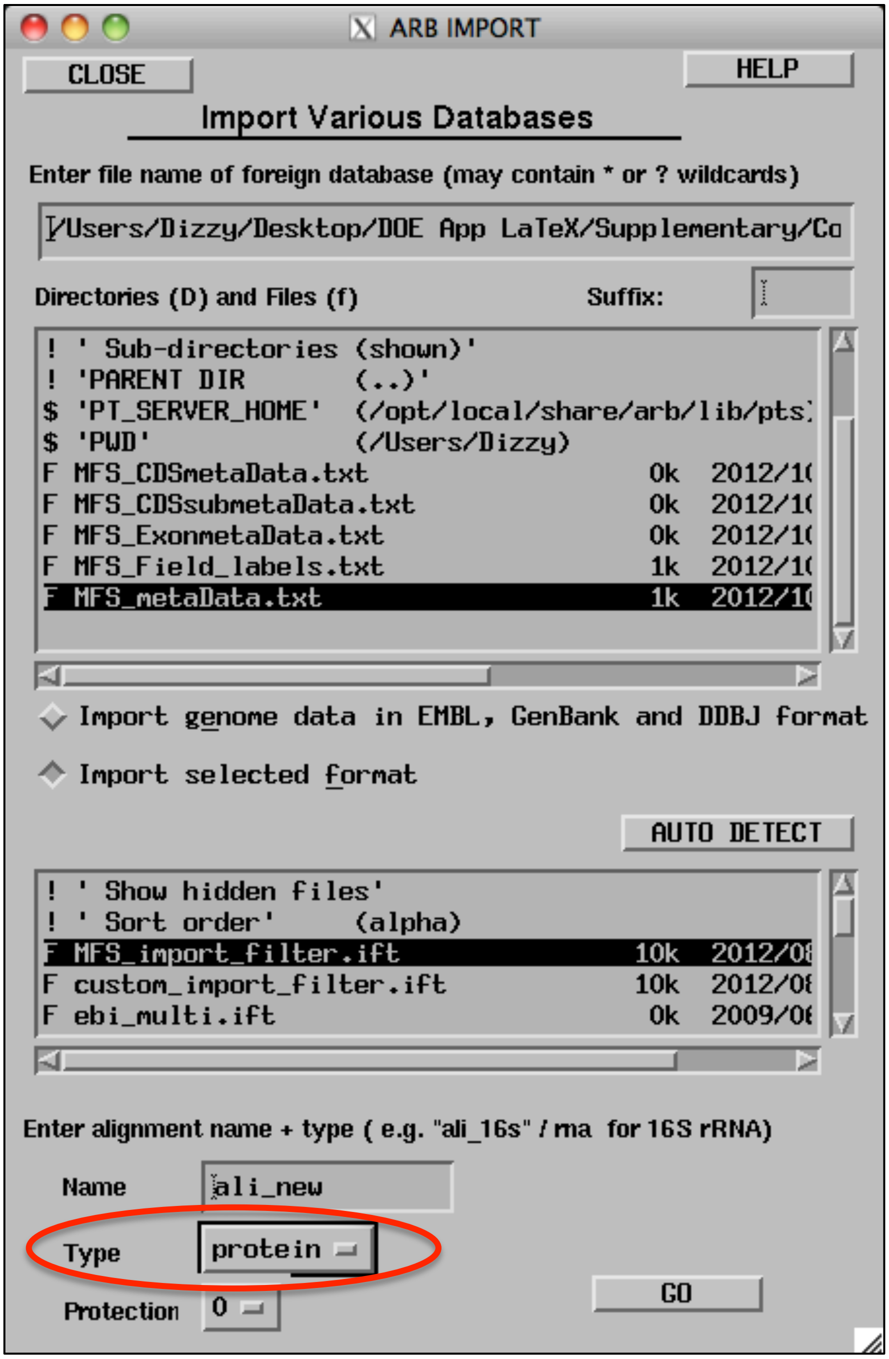

12. Verify the sequences/meta-data has been imported. Save the database as ***DB_Meta.arb.*** Close ARB.
    Note: You may need to select the list-view button to observe the list of all species.

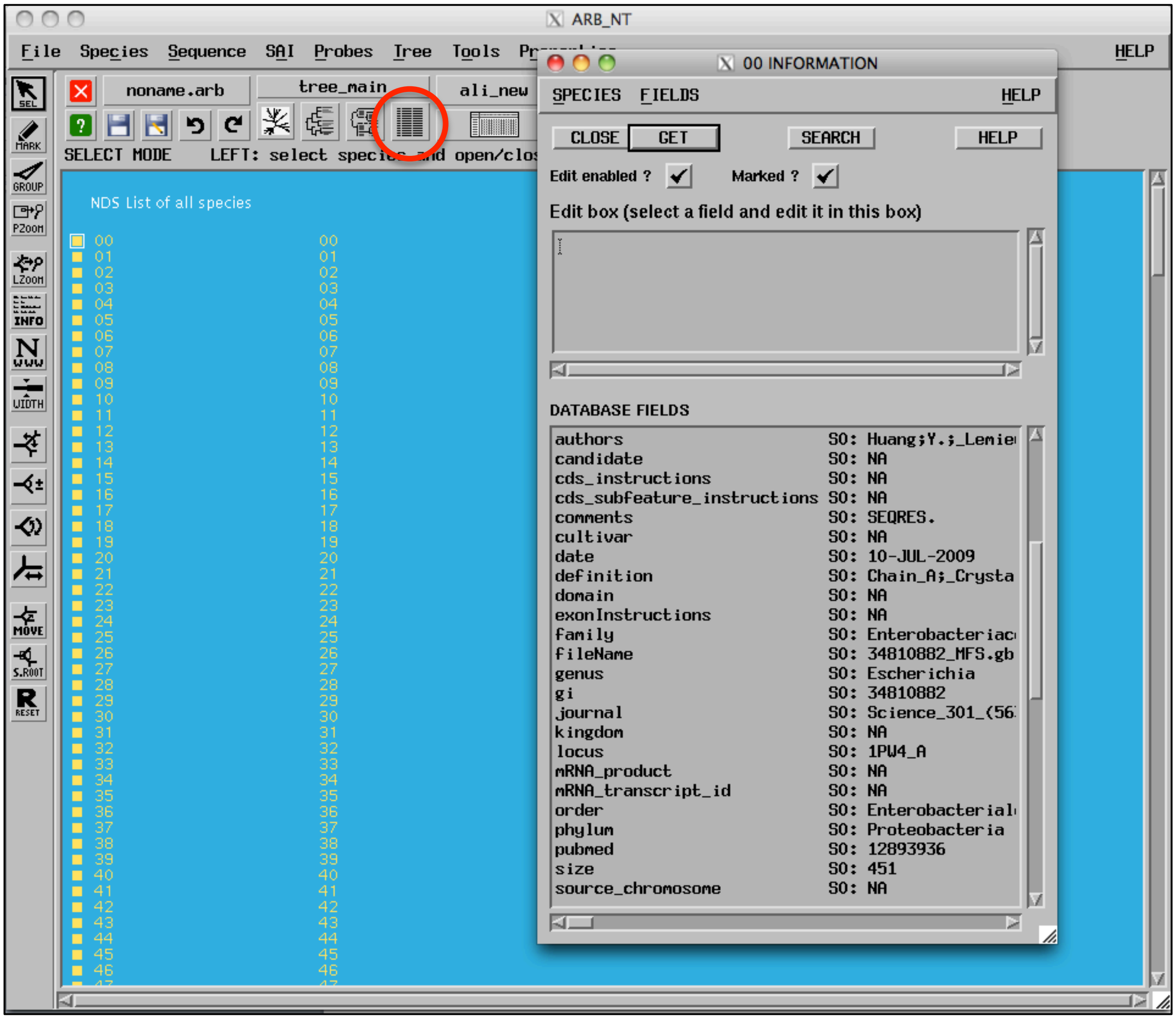

13. Open ARB. Import the alignment file. Use the ***fasta_wgap.ift*** filter this time. Change **Type** to **Protein**.
Note: If prompted, select 'Format (all)'. Also, if prompted choose 'Use found names'.

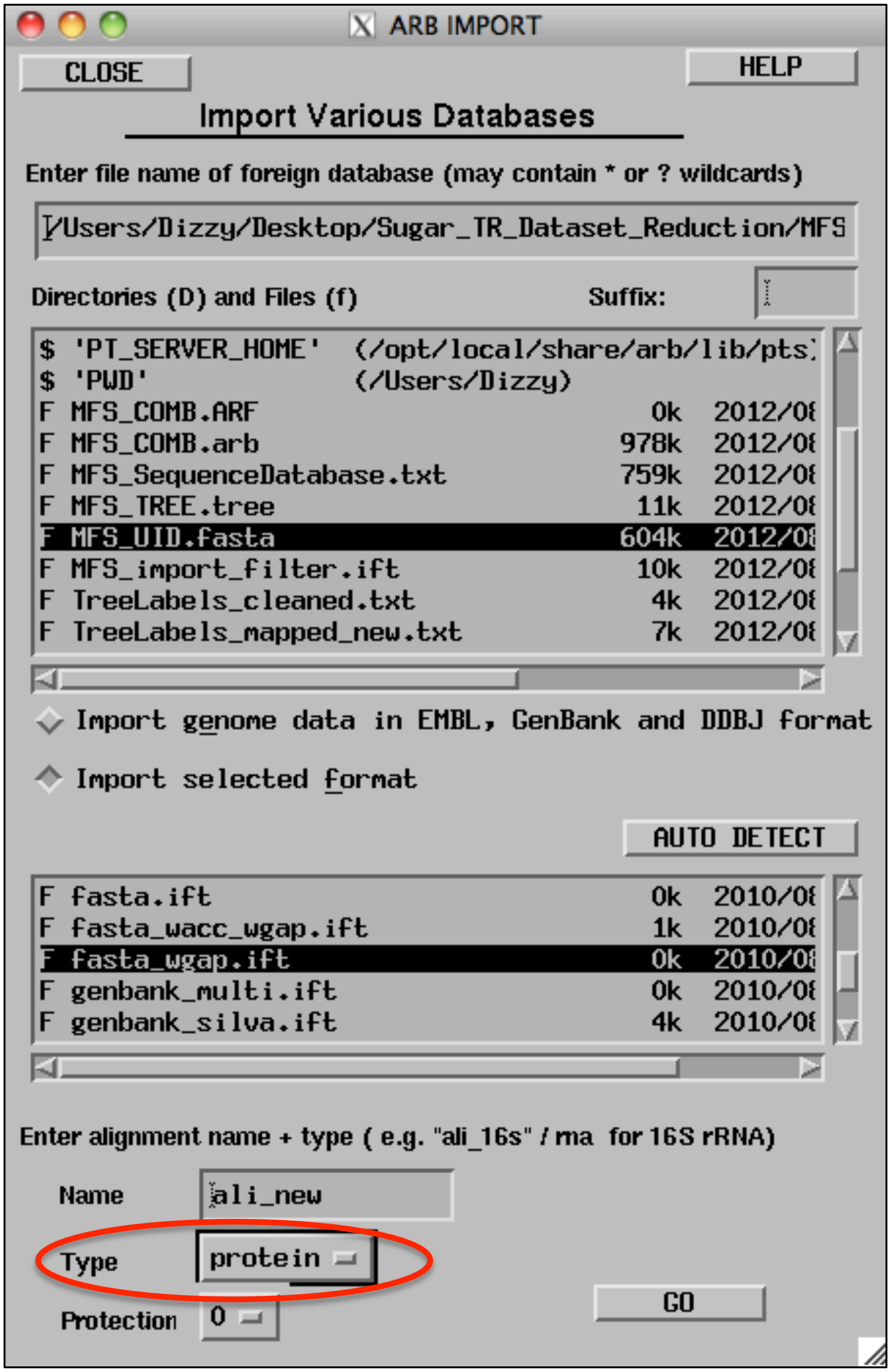

14. Create a new alignment field by copying ***ali_new***. Name the new field ***ali_ncbi***.

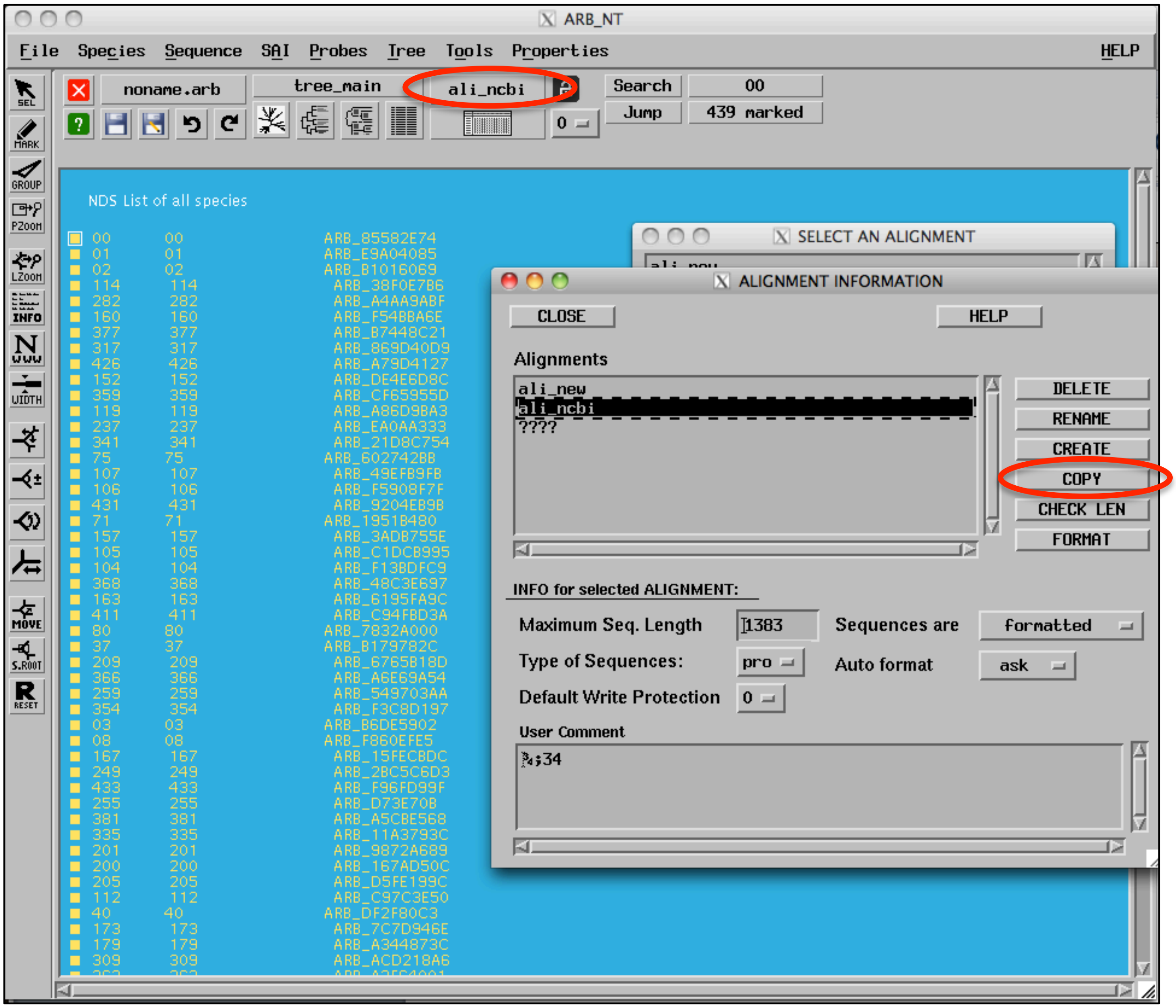

15. Save the database as ***DB_Aln.arb***.

16. Merge both ARB databases so that all meta-data, sequences and alignments are linked.
    a. Open ARB and select the merge database feature.
        i. Set database I to ***DB_Aln.arb***
        ii. Set database II to ***DB_Meta.arb***

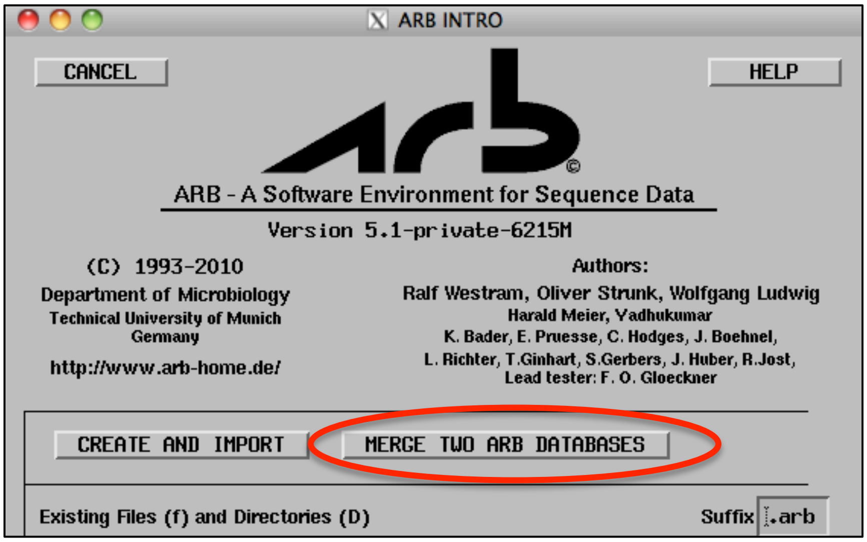

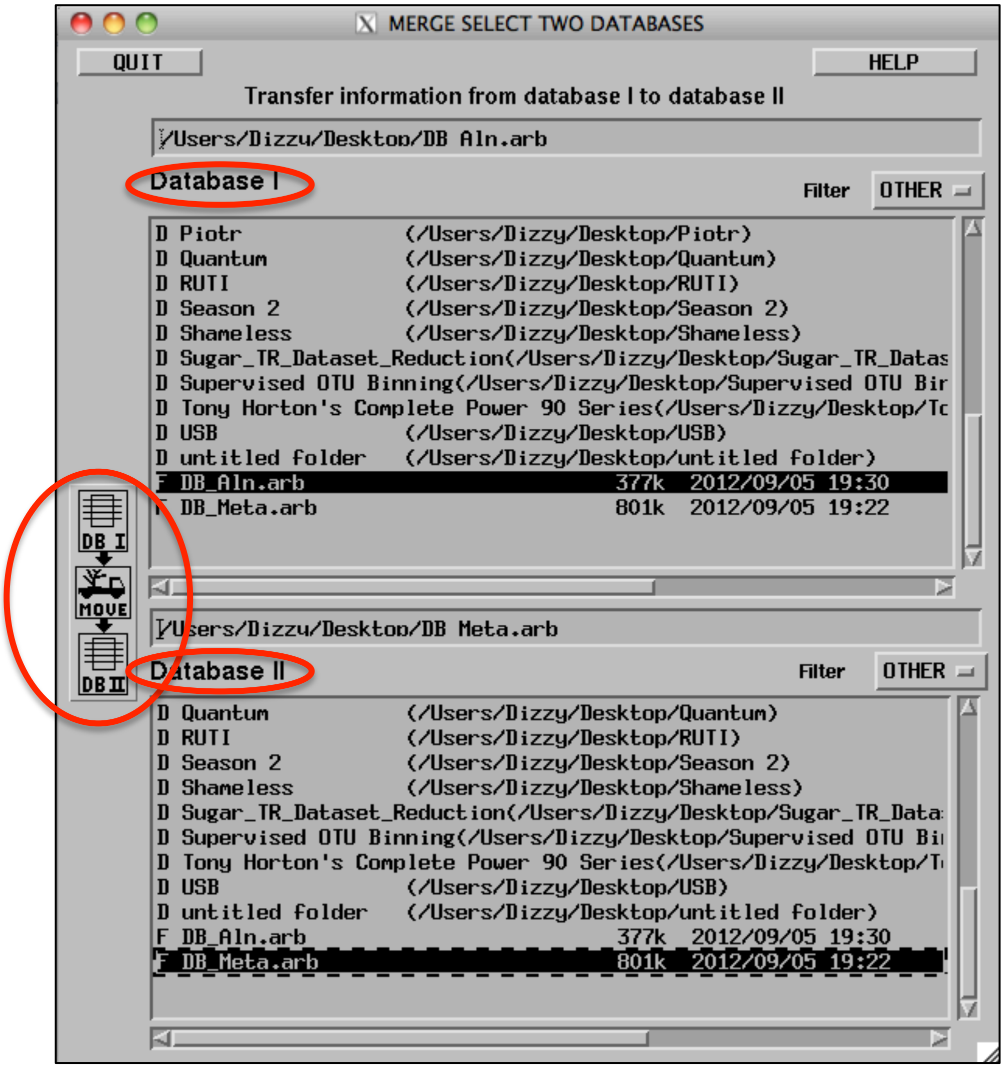

b. Check names...
    i. Check Allow Merging
    ii. Check Override
    Note: Do NOT click the Rename Species button.

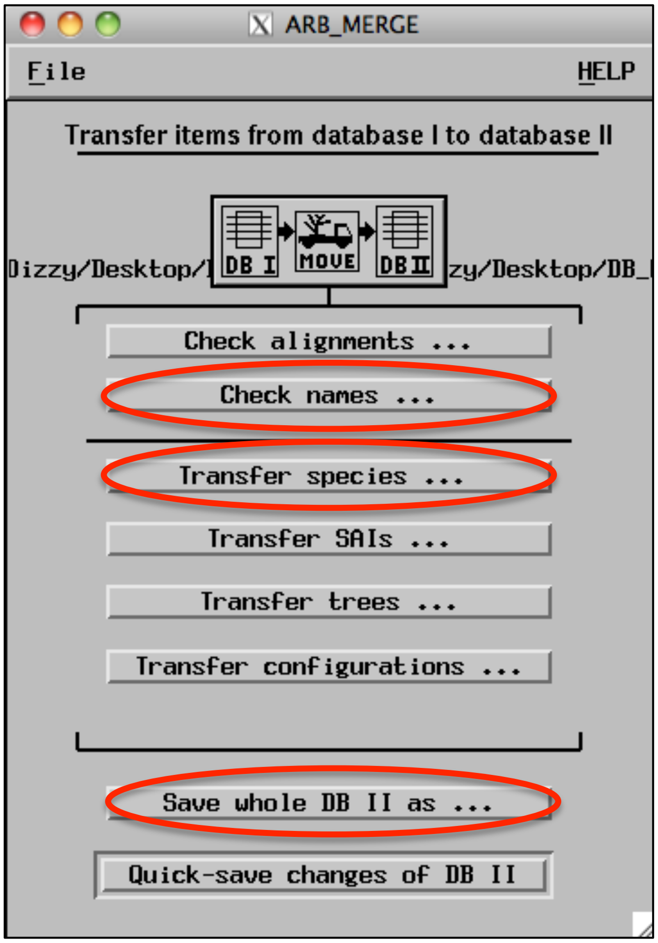

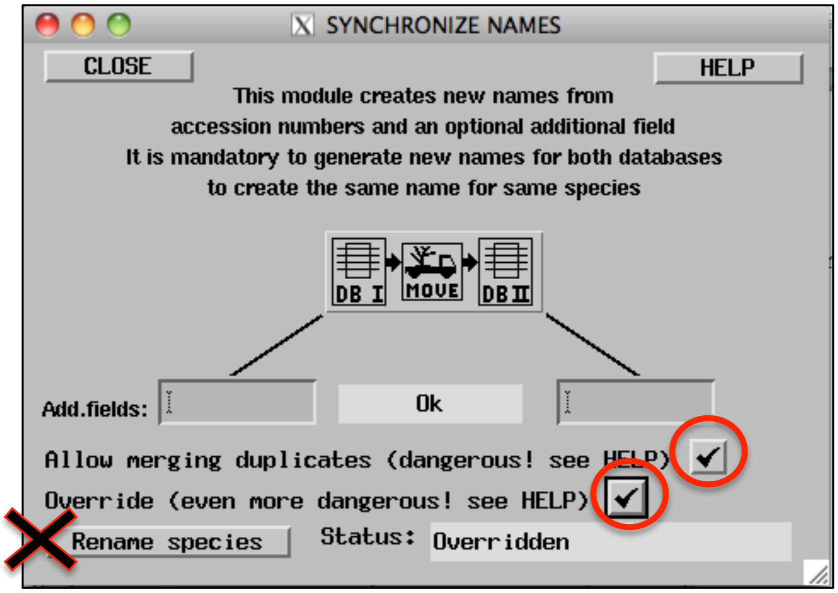

c. Transfer Species…
   i. Mark All Species in both DBI and DBII (via Search)
   ii. Click Transfer field of listed species…
   iii. Select name of alignment to transfer: ***aln_ncbi/data***
   iv. Click Go.

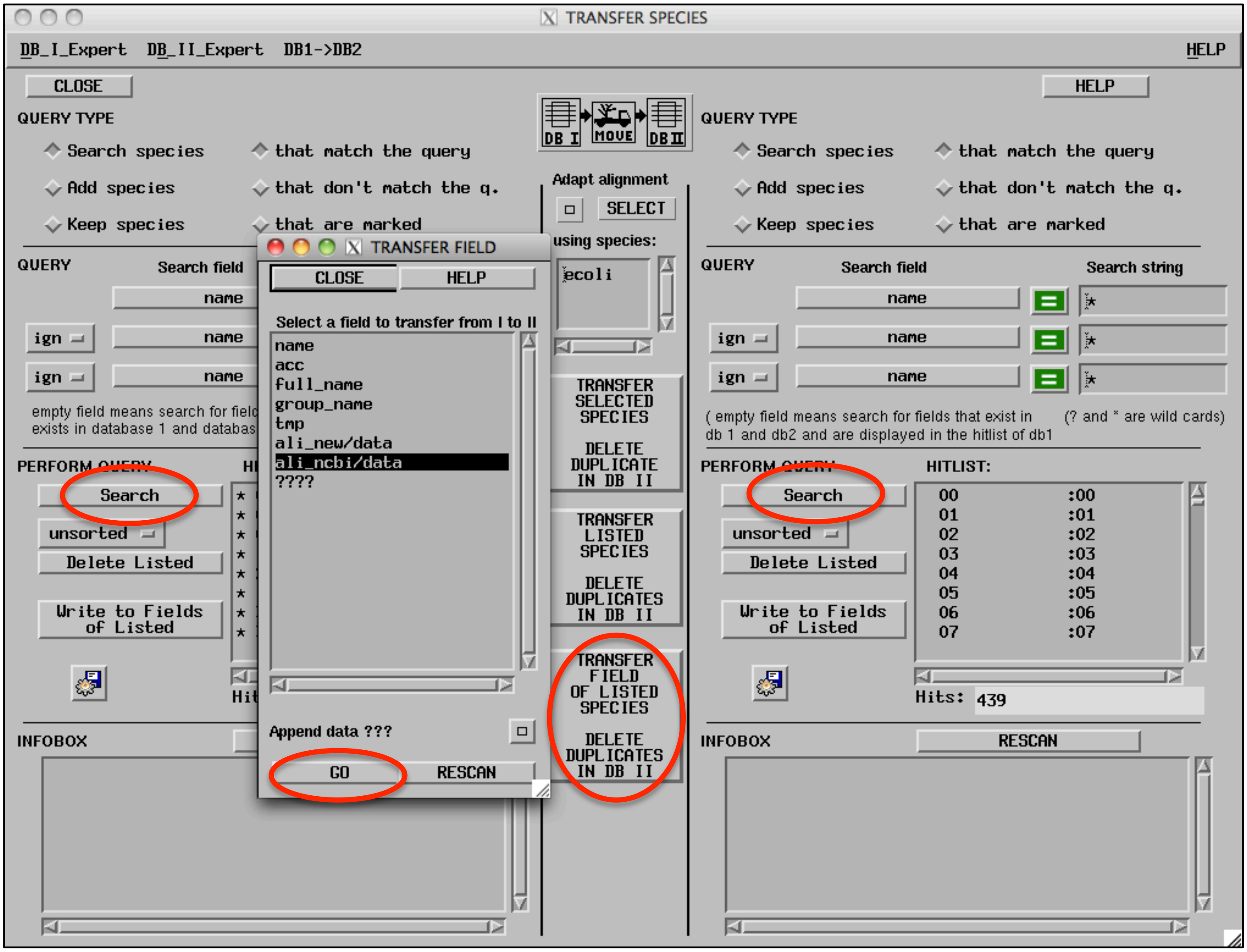

d. Close Transfer Species. Save the merged database as ***MFS_COMB.arb.***

17. Import tree to ARB
    a. Open ARB and the MFS_COMB database.
    b. Select Tree/Tree Admin/
    c. Import MFS_UID.tree from step 8.

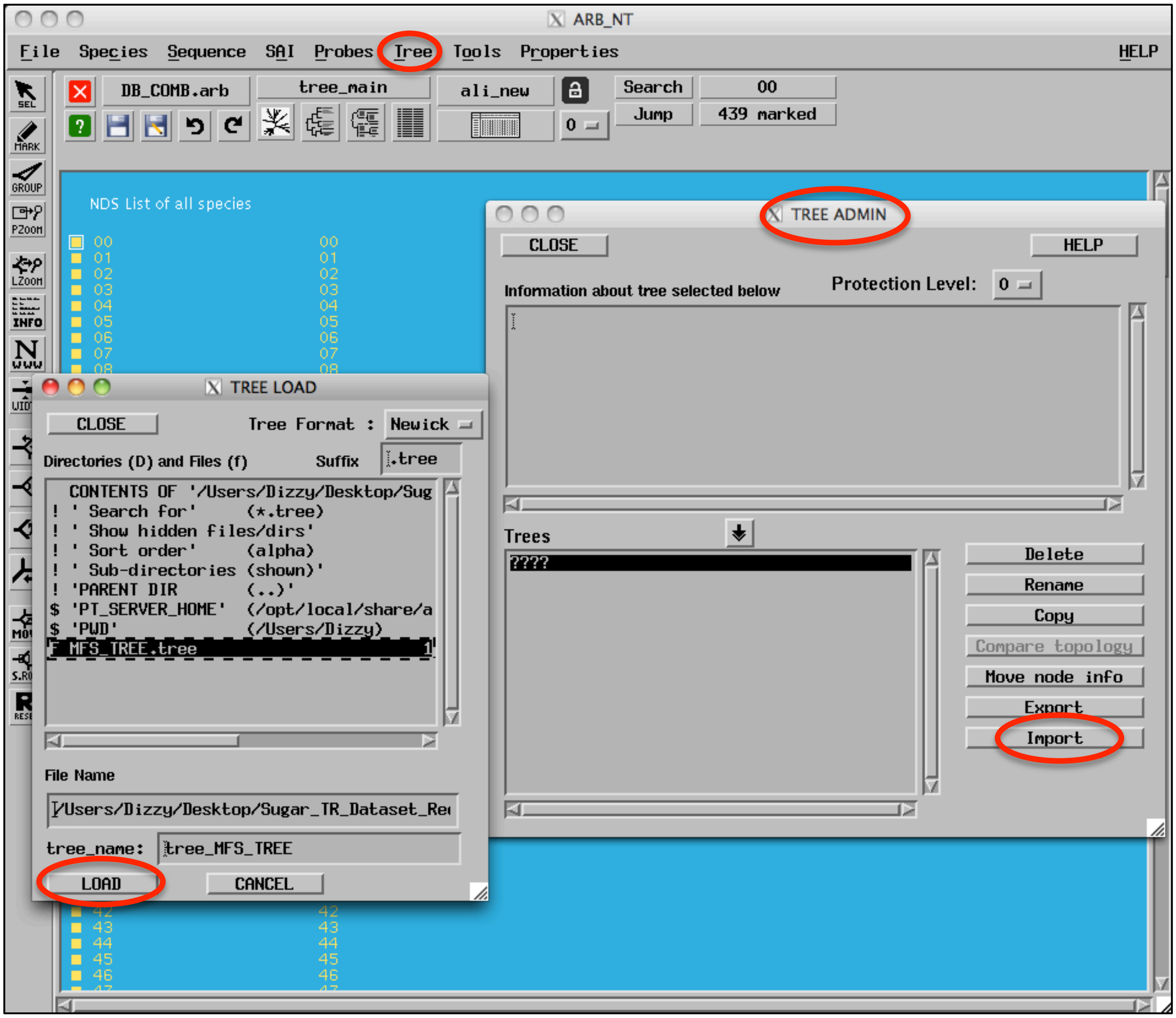

18. Save the database again so the tree is stored. The import process is complete!

Supplementary Material

Tree view using meta-label ***Family*** to investigate taxonomic relationship of leaves on tree. The leaf labels may be changed to display any of the meta-data choices via the Node Display Setup (NDS) under the Tree menu. To view different leaf labels on the tree go to: Tree → NDS (Node Display Setup).

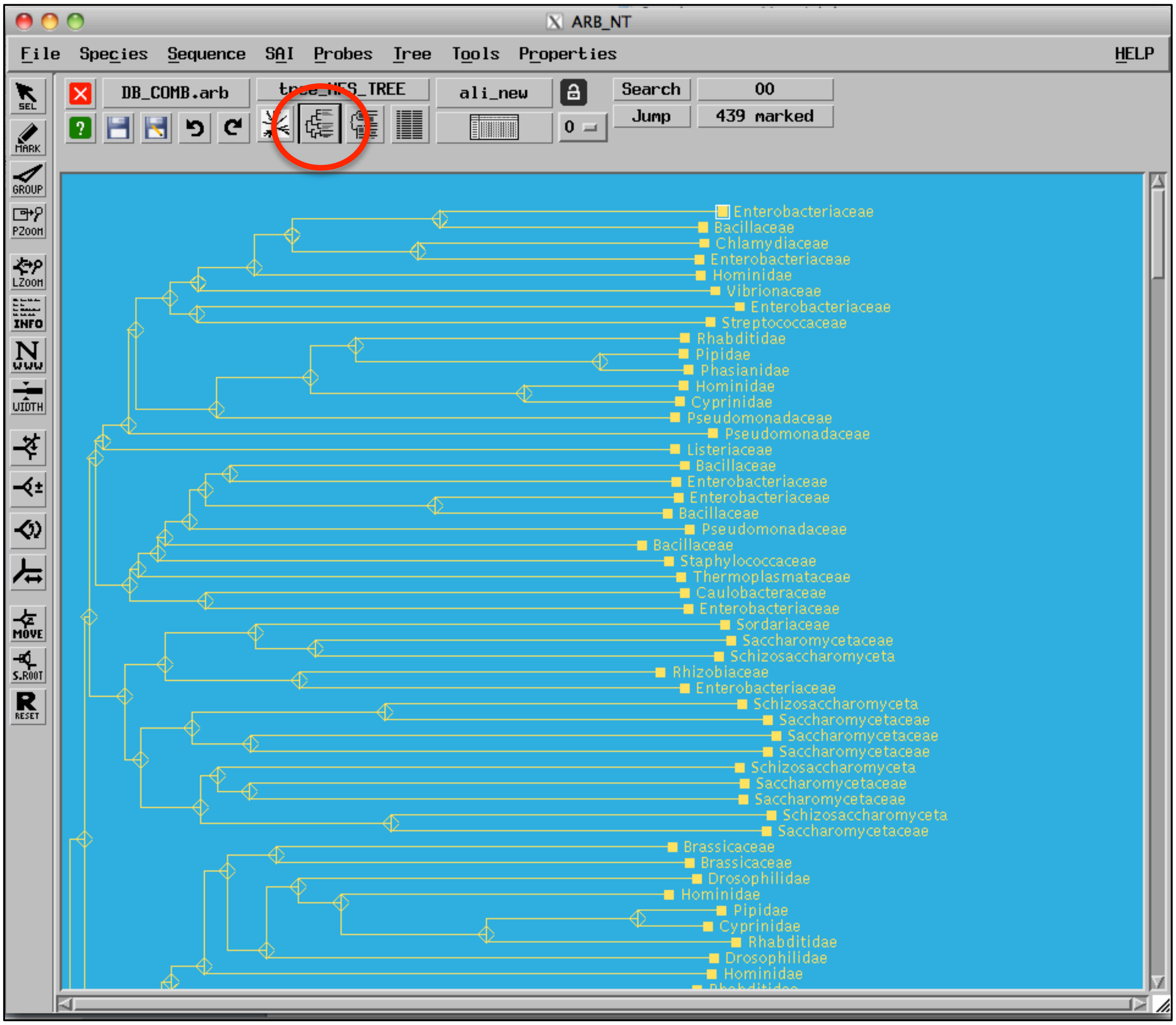

Supplementary Material

Appendix A - Renaming Tree Labels

The tree produced via the MFS CDD database contains leaves that are named using an index followed by an underscore followed by the identifier displayed on the tree (e.g. 2_2V8N|A). Note that the index is hidden when viewing the tree in ***CDTree***. We need to rename the leaves so that they match the unique identifier in our custom database (i.e. MFS_metaData.txt). This is necessary so that we can link the tree with the alignment and the meta-data once they are all imported into ARB.

Renaming the tree leaves:

1. Navigate to the 'Editing Leaf Labels for Tree MFS' page on the iTOL website as described in step 8, b, iii.
2. Highlight, then copy the current leaf labels on this page to a text file. Manually inspect this file to ensure that only leaf names have been copied. Delete anything else that was added to the file. Save this file as ***TreeLabels_Orig.txt***
3. The MFS CDD dataset has a slight naming inconsistency between the alignment identifiers and the tree leaves. The tree does not contain the '|' character, but for parsing purposes this must be inserted into the ***TreeLabels_Orig.txt*** file so that it is consistent with the alignment file. There are five instances where '|' must be inserted:

   0_1PW4|A   1_2GFP|A   2_2V8N|A   3_2CFQ|A   4_1PV7|A

4. Place ***TreeLabels_Orig.txt***, ***MFS_UID.fasta*** and ***MFS_Align.fasta*** in the same directory as ***rename_tree_leaves.py***. Run the ***rename_tree_leaves.py*** script to produce the new tree labels file, ***TreeLabels_Mapped_New.txt***. This file is formatted so that each line specifies one leaf name: the original followed by a tab and then the new one (i.e. the unique ID).

   python rename_tree_leaves.py -i MFS_Align.fasta -u MFS_UID.fasta -t TreeLabels_Orig.txt -o TreeLabels_Mapped_New.txt

5. Use the ***TreeLabels_Mapped_New.txt*** as directed in step 8, b, iii.
